# The Comparative Mortality of a Manufacturing and an Agricultural District

**Published:** 1855-12

**Authors:** J. Black


### THE COMPARATIVE MORTALITY OF A MANUFACTURING AND AN AGRICULTURAL DISTRICT.

By J. Black, M.D., F.G.S.

It is well known, from the Mortuary Registers, that there are considerable differences in the rates of mortality in the several districts of the kingdom; and that not only between communities widely situated from each other, but also between large districts in the same county. The principal causes recognised as producing this disparity of mortality, especially as it affects the comparative ages, are, the occupations of the people; their state in regard to habitations, numbers, residence in cities, towns or rural villages; and the position of these towns, whether in the hilly interior of the country or upon the airy coasts of the neighbouring sea.

Among these several causes tending to produce the different rates of mortality, there are none more productive of the unfavourable balance, than the crowded and often ill ventilated conditions of the factories and habitations of our manufacturing population, aggravated, as these evils are, by an ignorance and want of personal and domestic economy, occasioned greatly by the too early and continued employment of young and of married females in workshops and manufactories.

Even where the rate of mortality is shewn to be above the average of 23 in the 1000, it is seen, on examining the Register lists, that the excess is not generally found to exist at the ages above ten years, but in the years of infancy and childhood, *i.e.*, up to the fifth or sixth year—those periods of life where, upon the mother's nursing and care, the life, the ill health or death of the child depends. The too common ignorance and the want of these duties, among these classes of people, have, in many communities, produced an awfully premature destruction of human life, even to one half of all born alive, before the little victims have attained the fifth year of their age.

To illustrate the bearings of these different localities and modes of life in two districts, whose centres are within thirty miles of each other, I may cite the comparative rates of mortality, at two separate epochs of life, as they occur in the Registration Districts of Bolton-le-Moors and that of North Meols, in which last is situated the rapidly growing town of Southport, on the west coast of South Lancashire.

These returns were kindly furnished to me by the respective Superintendent Registrars; and I herewith extract the following results from them, as bearing chiefly on our present subject.

*Great and Little Bolton.*—The average number of deaths for each year of 1849 and 50 was 1770; and as the estimated population for the latter year was 59,050, this will give 1 in 33·62 of the population, or about 30 to 1000.

The average mortality for the two years, at and under five years of age, was 52·22 per cent. of the whole deaths; and 17·5 per cent. of the same, at and above sixty years of age, one person being registered as having died at 99.

*North Meols, including Southport.*—The population, calculated from the last census, was, in 1854, 8920, and in this year, 1855, at least 9000. The average number of deaths for each of the years 1853-54, is found to be 212; making 1 in 42·5 of the population, or at the ratio of 2·38 per cent., or 23·5 to 1000 living. The average mortality for each of the above years, at and under five years of age, was 38 per cent. of the whole deaths; and at and above sixty years of age, 23·1 per cent., the oldest person being registered as 95.

From the above comparative enumerations, it is seen how much different localities and social conditions affect the relative ratios of mortality. Though the districts under notice are not very distant, one of them is the site of a large manufacturing town—a type of many others, which are held to be unfavourable to early and advanced life; while the other district is a perfectly flat rural country, once an extensive *tarburium*, but since covered over with deep beds of fine marine sand, and later still by loam, and is generally cultivated. On the sea verge of this plain, about six miles in diameter, are situated the three towns of Southport, Churchtown and Crossens, all of which are included in the District Registration, now embracing, as noted above, fully 9000 inhabitants.

Though the meteorological conditions of the two districts in question are nearly alike, modified only by neighbouring hills in the one, and by the flat sea coast in the other, producing inequalities of temperature between them, the average for both places is nearly the same, and the clothing, diet and beverages of the people are alike.

When we come, however, to observe the relative rates of mortality in the aggregate, and at the several ages of life, we see differences, not so marked as in some other districts compared with each other, but sufficiently obvious; for, while the rate of mortality in Bolton is 30 in 1000 of the population, it is in North Meols only 23·5 in 1000—neither of them shewing the highest nor the lowest rate, according to Dr. Farr; for while in St. Saviour's, Southwark, 33 die out of every 1000, in Lewisham 17 only die in the 1000; while the average of all England is 23 in 1000, the mortality of males in Manchester being 37 in 1000.

The greatest disparity of ratio in the number of deaths, is seen at the several epochs of life; for while, in the Bolton district, from a careful examination of the Registers for 1849 and 50, as many as 52·22 per cent. of the whole deaths occur at and under five years of age, only 38 per cent. obtain in North Meols at and under the same age. This excess of infant mortality in Bolton takes place to about the same extent in Manchester and Liverpool; and it is a matter for serious consideration to all social economists, how to diminish or ameliorate it, however well acquainted all practical observers are with the causes producing the evil.

Again, while in Bolton 17·5 per cent. of the deaths occur at and above sixty years of age, in North Meols 23·1 per cent. happen at and above the same term of life.

From the respective data of the above two districts, we may be led to infer that the lives of infants and children are, in the one case, taken prematurely away from the toils and evils to come, and, on the other hand, are spared to augment the ratio of deaths in old age. However much truth there may be in these views, it is proper to observe that there are many invalid people, who come from the interior to recruit their healths in the more equable and temperate climate of Southport and Churchtown, and where several of them end their days, and so add a little to the relative numbers of deaths in advanced life.

Southport, November 1855.

---

